# Early Pleistocene cut marked hominin fossil from Koobi Fora, Kenya

**DOI:** 10.1038/s41598-023-35702-7

**Published:** 2023-06-26

**Authors:** Briana Pobiner, Michael Pante, Trevor Keevil

**Affiliations:** 1 Human Origins Program, Department of Anthropology, National Museum of Natural History, Smithsonian Institution, Washington, DC 20013 USA; 2 Department of Anthropology and Geography, Colorado State University, Fort Collins, CO 80523 USA; 3 Department of Anthropology, Purdue University, West Lafayette, IN 47907 USA

**Keywords:** Archaeology, Biological anthropology

## Abstract

Identification of butchery marks on hominin fossils from the early Pleistocene is rare. Our taphonomic investigation of published hominin fossils from the Turkana region of Kenya revealed likely cut marks on KNM-ER 741, a ~ 1.45 Ma proximal hominin left tibia shaft found in the Okote Member of the Koobi Fora Formation. An impression of the marks was created with dental molding material and scanned with a Nanovea white-light confocal profilometer, and the resulting 3-D models were measured and compared with an actualistic database of 898 individual tooth, butchery, and trample marks created through controlled experiments. This comparison confirms the presence of multiple ancient cut marks that are consistent with those produced experimentally. These are to our knowledge the first (and to date only) cut marks identified on an early Pleistocene postcranial hominin fossil.

## Introduction

While it is assumed that Pliocene and early Pleistocene hominins were sometimes the victims of predation by the many taxa of larger carnivores with which they coexisted, taphonomic evidence for such interactions in the form of carnivore chewing damage or tooth marks on hominin fossils is relatively uncommon. In 2011, Hart and Sussman^1^ listed 10 hominins dated to between 6 million years ago and 50,000 years ago with evidence of terrestrial carnivore or raptor predation; this list does not include carnivore damage on *Australopithecus anamensis* fossils from Kanapoi and Allia Bay, Kenya^2,3^ and *Australopithecus africanus* fossils from Member 4 of Sterktfontein, South Africa^4^; a tooth mark on the pelvis of the AL 288–1 (“Lucy”) *Australopithecus afarensis* partial skeleton from Hadar, Ethiopia (^5^, though see^6^ for an alternate interpretation of this mark); tooth marks on the *Paranthropus robustus* SK 54 cranium from Swartkrans, South Africa^7^; and at least two *Homo habilis* specimens from Olduvai Gorge, Tanzania with evidence of crocodile predation^8^.

**Figure 1:**
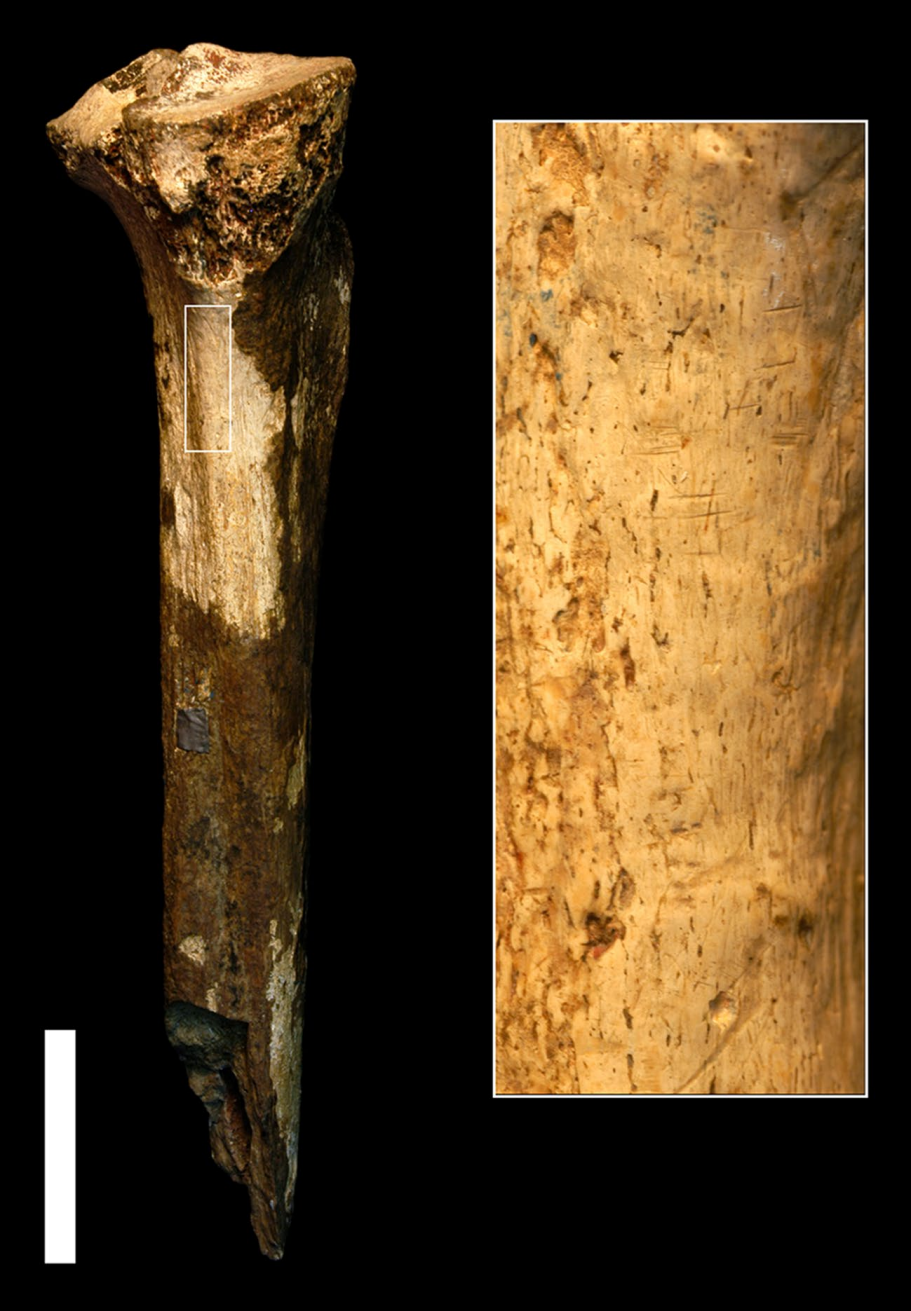
Complete view of tibia (KNM-ER 741) and magnified area that shows cut marks perpendicular to the long axis of the specimen. Scale = 4 cm.

In July 2017, one of us (Pobiner) undertook a pilot study of the taphonomy of published hominin postcranial fossils from the Turkana region of Kenya dated to ~ 1.8 to 1.5 Ma, with an expectation of potentially finding some carnivore damage on these fossils. However, she unexpectedly observed potential butchery marks on a single fossil: KNM-ER 741 (Fig. 1). This observation was unexpected because while butchery marks left by hominins on animal fossils beginning by at least the early Pleistocene point to increased meat and marrow acquisition during the evolution of the genus *Homo*^9–12^ and hundreds of cut marked fossils of other animals have been identified from the Okote Member of the Koobi Fora Formation^13–15^, no cut marks on hominin fossils from this temporal and geographic area have been reported.

In 1970, Mary Leakey found the proximal half of a left tibia, KNM-ER 741^16,17^. A surface discovery, Leakey retrieved the fossil from exposures of the Okote Member, Koobi Fora Formation in Area 1 of the Ileret region. While 15 additional hominin specimens were found during the same research expedition in 1970, no additional information is available regarding the discovery of this fossil and the information about it in the Koobi Fora monograph does not specify whether other hominin fossils were found nearby. Its stratigraphic position is noted as “Ileret member, *c.* 2–4 m above the top of the Lower/Middle Tuff (collection unit 5)” with a depositional environment of “Fluvial, probably the edge of a channel” and additional notes that “The sand matrix on KNM-ER 741, a surface find, indicates association with the channel lens rather than more proximal fine-grained sediments” (^16^^,^^17^: 110). Anna K. Behrensmeyer’s unpublished field notes from her 1973 documentation of the context of the site indicate that some faunal specimens were found in the general vicinity of the KNM-ER 741 discovery; a few may have been collected. Her field notes include a description of the surfaces of the tibia as relatively fresh with slight weathering of the bone grain, fine longitudinal cracking which could be pre-depositional weathering, and no major cracks. She observed some dissolution and whitening of the surface, some sand was in the trabeculae exposed along the edge of the proximal articulation, the marrow cavity was hollow without matrix, and the distal break (on the midshaft) had both fresh and ancient fractures (A.K. Behrensmeyer, *pers. comm.*).

KNM-ER 741 was originally attributed to *Australopithecus boisei* when it was first published by Richard Leakey^16,17^ and then described by Leakey and colleagues^18^. Two decades later, Alan Walker revised the taxonomic attribution of this specimen to *Homo erectus* based on comparison with KNM-WT 15000, the “Turkana Boy” or Nariokotome partial juvenile skeleton^19^. The entry about KNM-ER 741 in the Wiley-Blackwell Encyclopedia of Human Evolution says “current conventional taxonomic allocation is *H. erectus* or Hominin gen et sp. indet.” and “because so little is known about the tibial morphology of early hominins other than *Australopithecus afarensis* it may be premature to rule out the possibility that it belongs to *Homo habilis* or *Paranthropus boisei*”^20^. Due to the taxonomic uncertainty of this fossil, we simply refer to it in this study as a hominin (hominin gen. et sp. indet). The age of the fossil is estimated to be about 1.45 Ma^20^.

While examining KNM-ER 741 at the National Museums of Kenya Nairobi Museum, Pobiner identified a series of bone surface modifications on the medial side of the proximal shaft which appeared consistent with stone tool cut marks (Fig. 1). Pobiner created an impression of 11 of these marks with dental molding material. After she returned to the US, Pobiner sent the molds to Pante and Keevil for analysis and comparison to marks on modern bones made by known processes without any contextual or other information about the bone the marks were on, although Pante knew that she had been doing research at the National Museums of Kenya.

This fossil specimen is a nearly complete proximal tibia with the proximal end, proximal shaft, and midshaft present. The specimen has a transverse and longitudinal (not spiral) ancient and modern fracture on the distal shaft. Most of the surface of this fossil specimen is darkly colored (likely manganese stained) with a poorly preserved, rough appearing cortical surface due to weathering, abrasion, and/or some other physical or chemical process. In the original description of the fossil, it is noted that “the margins of the articular surfaces have been abraded” (^16^^,^^17^:110). However, the relatively small area where these marks occur is yellow-whitish colored with very good cortical bone surface preservation. No other bone surface modifications were observed on any other parts of the specimen outside of this area. No information on whether this specimen has undergone any preparation since it was discovered exists, though no preparation or excavation damage was detected. The marks observed and described here are the same color as the rest of the bone surface, so they are unlikely to be modern damage. No cleaning of the marks or any other preparation of this fossil was carried out during this study.

## Results

**Figure 2:**
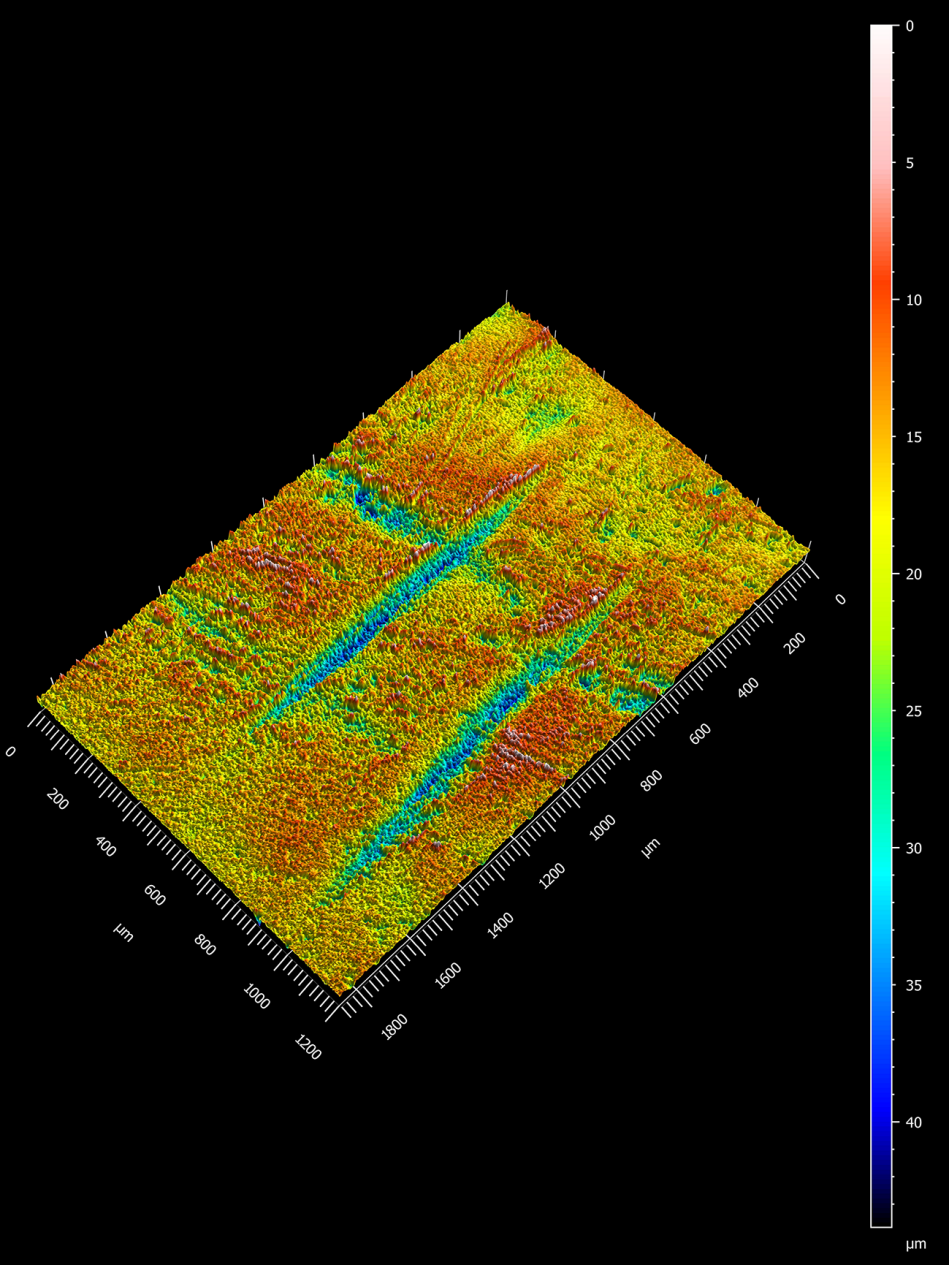
3D model of marks 7 and 8 identified as cut marks by the quadratic discriminant model.

The majority of the bone surface modifications identified and studied here are short, narrow linear marks with a straight trajectory and a closed-V-shaped cross section, oriented in the same direction: transverse and slightly oblique to the long bone axis. The V-shape of these marks and their straight trajectories are strong indicators that they are cut marks^21^. They are not shallow, fine, randomly oriented striae which are randomly distributed on various parts of the fossil; these features are characteristics of sedimentary abrasion^21^. The marks also do not look similar to other non-anthropogenic agents which can inflict linear marks, such as insects, plant roots, or raptor beaks^22^. While internal striations were not observed, this feature can be difficult to observe without higher (at least 40×) magnification. Shoulder effects were observed on some of the marks (Fig. 2). Flaking effects were not observed on these marks. Flaking effects are more likely to occur with retouched versus unretouched flakes^21^, and in our experience are less often identified on fossilized bones when compared to modern butchered bones. These marks all occur in the same general area on the shaft of the bone; some are isolated while others occur in groups, adjacent to each other in patches. While it is possible that the marks occurred recently (after the bone was fossilized), we think this is unlikely because the color of the marks is not different from the color of the area of the bone on which they occur, and the marks are neither randomly oriented nor randomly distributed.

**Figure 3:**
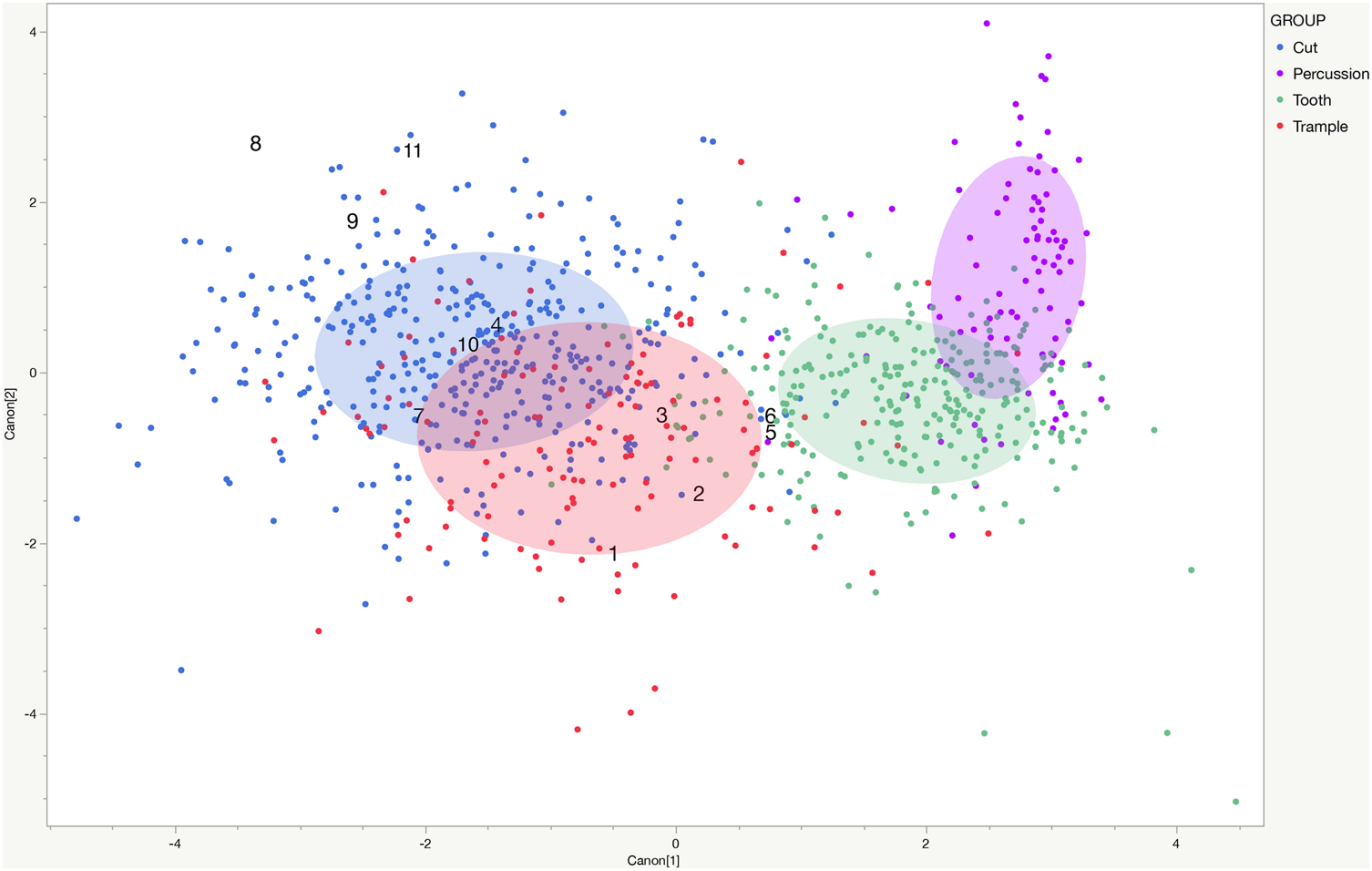
Results of quadratic discriminant analysis. Archaeological specimens are identified by their given ID numbers. Circles encompass 50% of the data for each mark type. The model is shown in two dimensions here, but has a third canonical dimension.

**Figure 4:**
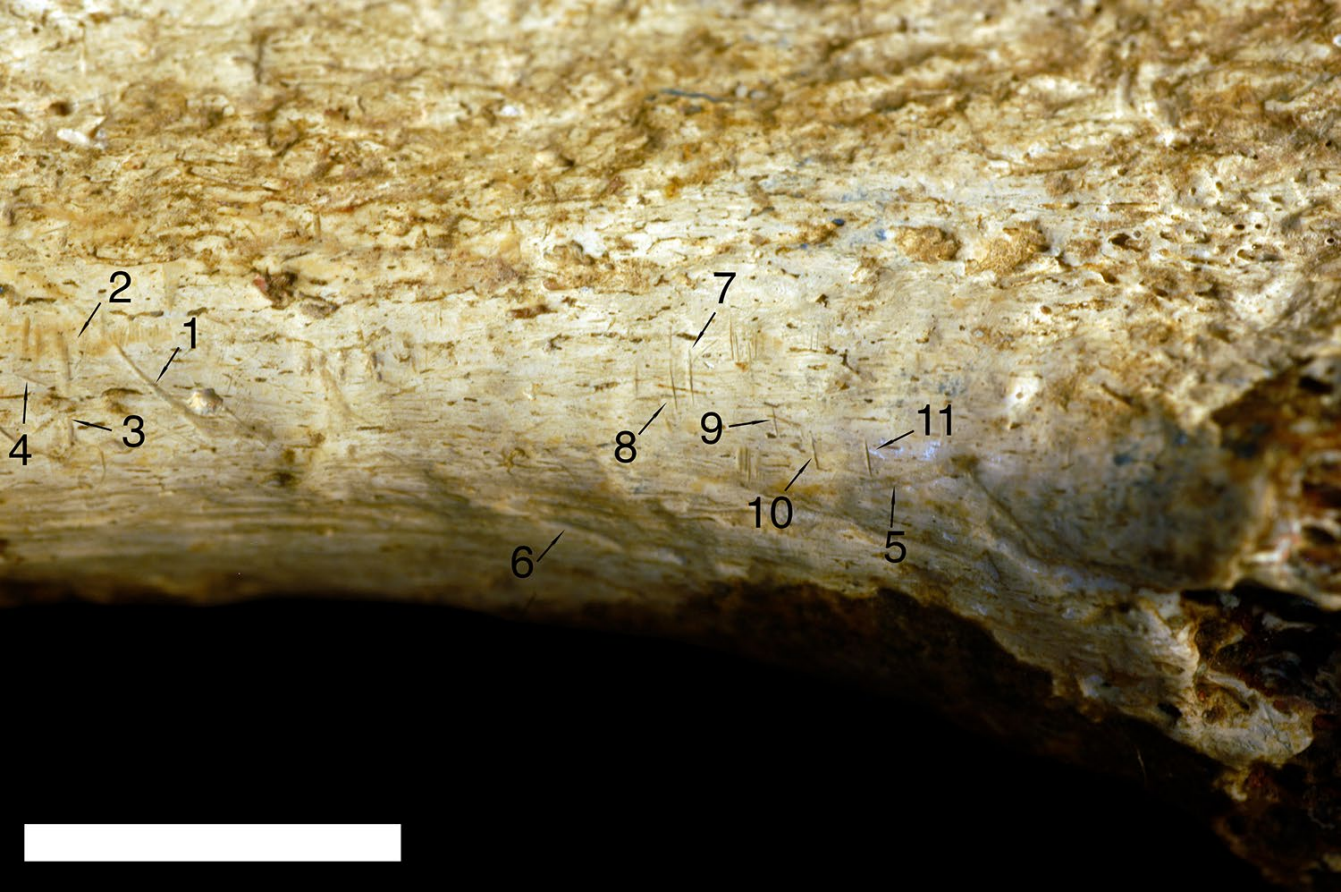
Nine marks identified as cut marks (mark numbers 1–4 and 7–11) and two identified as tooth marks (mark numbers 5 and 6) based on comparison with 898 known bone surface modifications using a quadratic discriminant analysis of the micromorphological measurements collected in the study. Scale = 1 cm.

**Figure 5:**
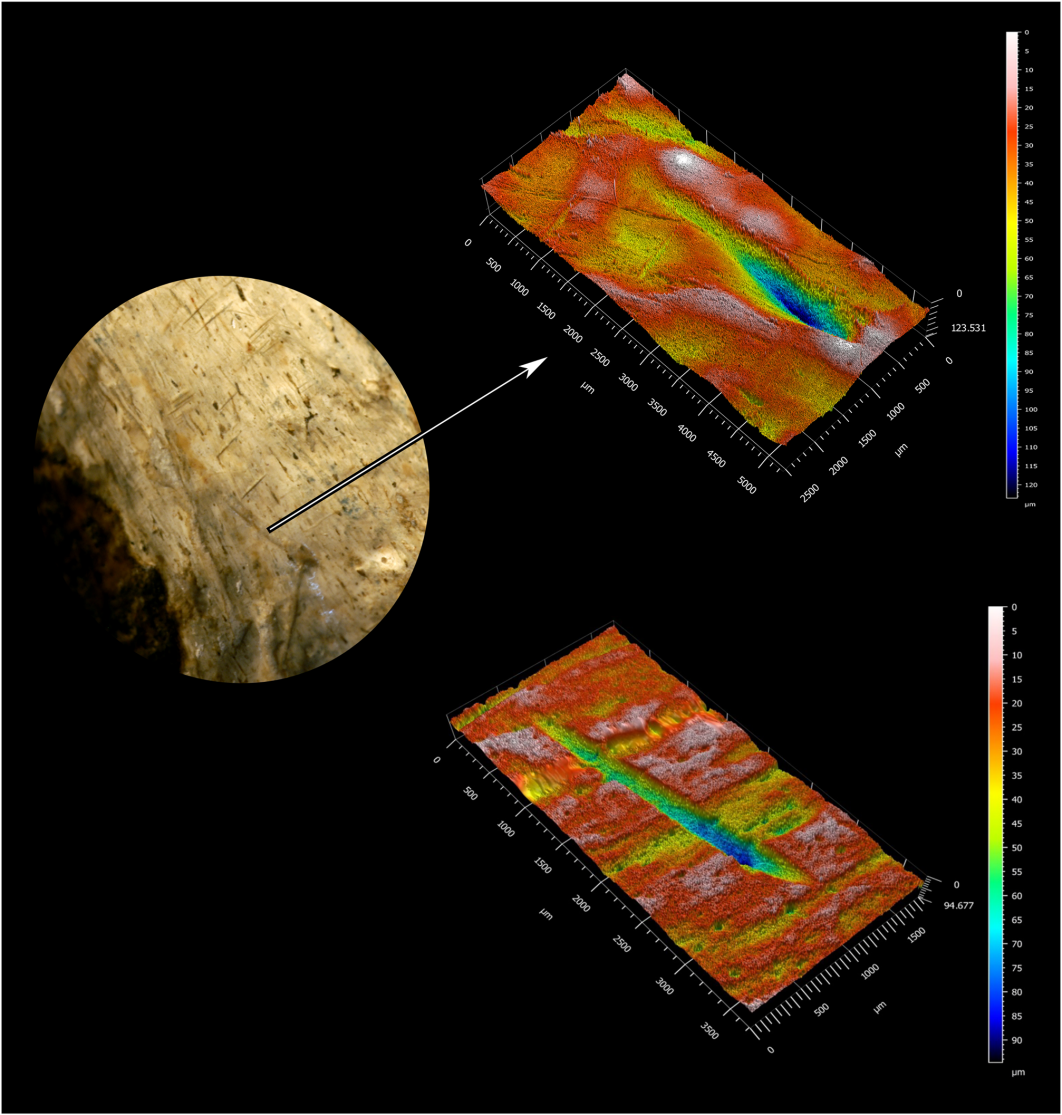
A closeup of mark 5 and the processed 3-D model compared with a modern lion tooth mark.

Of the 11 marks measured, 9 were classified as cut marks and 2 as tooth marks (Table 1, Figs. 3 and 4). Six of the cut marks were classified with posterior probabilities near or above 90% indicating a high level of confidence in the identification (see Fig. 3 for an image that identifies all of the analyzed marks). This accords with the qualitative observations and descriptions of the marks above. The two marks classified as tooth marks were compared with 163 known marks inflicted by bone crunching carnivores (Hyaenidae, Canidae, Crocodylidae), and 58 known marks from flesh specialist carnivores (Felidae). The quadratic model can discriminate between bone crunching carnivores and flesh specialist felids (only represented by African lions) with 77% accuracy. Results show both marks classify with marks made by flesh specialists with posterior probabilities of 100% (see Fig. 5 for a comparison between mark 5 and a modern lion tooth mark).

**Table 1:** Mark identifications and posterior probabilities.

Results of QDA				Measurements from 3D models						Measurements from profiles					
Mark ID	QDA identification	Probability (Predicted)	Secondary identifications	Surface area (µm^2^)	Volume (µm^3^)	Maximum depth (µm)	Mean depth (µm)	Maximum length (µm)	Maximum width (µm)	Maximum depth (µm)	Area (µm^2^)	Width (µm)	Roughness (RA)	Angle (°)	Radius (µm)
1	Cut	0.89	Trample 0.11	893,975	9,200,199	27.4	10.3	2957.8	588.6	28.8	3555.7	330	1.1	161.6	997.2
2	Cut	0.59	Tooth 0.39	417,500	4,792,604	29.1	11.5	1490.6	309.4	20.5	2808.9	260	0.6	165.9	640.6
3	Cut	0.91	Tooth 0.10	187,175	1,997,084	25.9	10.7	1109.1	231.5	19.4	2123.6	220	1.3	163.7	546.1
4	Cut	0.67	Trample 0.33	225,050	5,158,590	50.6	22.9	4807.9	284.9	30.5	2753.3	190	1.7	151.5	233.1
5	Tooth	0.65	Cut 0.32	2,069,050	77,950,000	95.8	37.7	3922.0	704.7	80.2	22,083.8	579	0.9	146.1	571.2
6	Tooth	0.95		2,335,300	92,340,000	86.8	39.5	4791.7	749.3	74.2	26,670.8	670	1.8	159.8	1092.7
7	Cut	0.95	Trample 0.13	111,400	897,792	21.1	8.1	1599.4	143.1	16.6	663.1	95	1.4	145.0	96.3
8	Cut	0.85	Trample 0.15	48,900	297,642	16.2	6.1	1522.4	80.2	17.2	650.1	75	1.0	126.7	107.2
9	Cut	0.92	Trample 0.20	55,450	324,502	17.6	5.9	947.7	97.5	17.4	687.7	85	0.8	120.9	120.8
10	Cut	0.99		65,775	795,333	24.2	12.1	990.9	184.7	13.3	710.9	100	1.4	144.9	165.5
11	Cut	0.61	Trample 0.39	44,325	627,685	29.3	14.2	900.6	105.1	24.0	1058.4	85	1.0	96.5	54.5

## Discussion

### Human anthropophagy: definitions and taphonomic criteria

Anthropophagy is often used synonymously with cannibalism, but the former can be defined as occasional consumption of humans by other humans while the latter usually implies a cultural practice^23^. Cannibalism is defined as the act of consuming tissues of individuals of the same species^24^ and occurs in over 1300 species of animals in the wild, including several primates^25^. In the case of this hominin fossil tibia, we do not know the identity of the species of the consumer (the species which inflicted the butchery marks) nor the consumed (the species of the hominin tibia). Because of this lack of information, we cannot make a claim of cannibalism based on the evidence presented here. However, due to the possibility that an individual of the same species of hominin to which the tibia belongs also inflicted the cut marks on the tibia, we include a discussion about hominin anthropophagy, which can include cannibalism.

Previous researchers have defined and described the various motivations or contexts for human cannibalism in different ways, including: survival; gastronomic or dietary; aggressive; psychotic or criminal; warfare; affectionate; funerary, ritual, spiritual, or magical; and medicinal (e.g.,^26^^,^^27^^,^^28^^,^^29^^,^^30^^,^^31^). These types of cannibalism have also sometimes been subdivided into social divisions that include *aggressive* (consuming enemies) versus* affectionate* (consuming friends or relatives), or *endocannibalism* (consumption of individuals within the group, usually associated with sacred beliefs and spiritual regeneration of the deceased) versus *exocannibalism* (consumption of outsiders, usually associated with hostility and violence)^26^.

Biological anthropologists and archaeologists have generated taphonomic criteria for different kinds of cannibalism to better understand the intent of anthropogenic modification of hominin skeletal remains. These include abundant anthropogenic modifications on more than 20% of human remains; intensive processing of bodies; greater abundance of cut marks related to defleshing and filleting than dismembering; the presence of human tooth marks or chewing damage; and the similar treatment of human and animal remains^23,26–31^. Differentiating between nutritional and ritual cannibalism is primarily based on a comparison of the taphonomic traces on and post-processing discard patterns of hominin and non-hominin remains^24,25,28,29,32,33^. Evidence for marrow and brain extraction are additional indications of nutritional cannibalism, while ritual cannibalism might be inferred in instances of defleshing without marrow extraction^25^. Saladié et al.^33^ recently found that high frequencies of anthropogenic modifications (> 30%) are common after an intensive butchering process intended to prepare a hominin body for consumption in different contexts, contradicting previously held assumptions about cut mark intensity. A full discussion of these past behaviors and the criteria used to identify them are outside the context of this study, though they are briefly summarized here; in order to apply these criteria, richer contextual evidence than what was found with KNM-ER 741 is necessary.

A more recent study by Bello et al. of four archaeological sites (one interpreted as cannibalism and three interpreted as funerary defleshing and disarticulation after a period of decay) determined that a distinction between butchery marks reflecting cannibalism of fresh bodies and secondary burials of bodies at various stages of decomposition can be made based on frequency, distribution, and micromorphometric characteristics of cut marks^34^. This study concluded that cannibalized sites exhibit higher frequencies of cut marks overall, but especially disarticulation cut marks on both persistent and labile joints, while secondary burial sites exhibit low frequencies of cut marks on labile joints because they have been naturally disarticulated over time. Bello et al. found that disarticulation cut marks at the cannibalized site (Gough’s Cave, UK) were deeper and wider than filleting marks at all four archaeological sites, but that filleting marks on long bones associated with larger muscles (humerus, femur, and tibia) were wider and deeper than those on other long bones (radius, ulna, and fibula). They also found that cut marks on the bones from the funerary defleshing sites (Lepenski Vir, Padina, and Vlasac, Serbia) were wider and deeper than cut marks found on other butchered animals at the site, implying the use of more strength to deflesh human bodies, possibly due to “the difficulty in removing remnant tissue on partially desiccated remains” (^34^:739).

Given the lack of evidence in the Early Pleistocene for primary or secondary burial, or other ritual behaviors, we think only one of the three functional types of human cannibalism outlined by Fernández-Jalvo et al.^26^ is potentially applicable to this study: nutritional cannibalism. Nutritional cannibalism occurs for the sole purpose of obtaining food and can be divided into two categories: (1) *incidental* cannibalism, which is focused on survival; this occurs in periods of food scarcity or due to catastrophes, i.e., is starvation-induced; (2) *long duration* cannibalism, which is also called gastronomic or dietary cannibalism; humans are simply part of the diet of other humans.

Features of human tooth marks on bones have been identified through experimental studies and applied to some fossil assemblages with other evidence of cannibalism (e.g., ^22^^,^^35^^,^^36^). Fernández-Jalvo and Andrews^35^ identified bent ends/fraying on thin bones such as ribs and vertebrae apophyses; crenulated or chewed edges, often with a double arch puncture; tooth punctures usually left by molars or premolars which are most often triangular, dispersed, small (< 4 mm), and occur infrequently; and shallow linear marks left by incisors which are usually superficial, less deep and distinct than carnivore tooth scores, and not easily distinguishable without magnification. Saladié et al.^36^ further identified furrowing and scooping out of long bone epiphyses; crenulated and saw-toothed edges on flat bones, sometimes associated with notches; longitudinal cracks; crushing; peeling and bent ends; and pits, punctures, and scores. In their study, tooth punctures and pits tended to have irregular contours, including crescent shapes; tooth scores were primarily shallow, some with flaking on the shoulder or bottom of the groove, and a few with microstriations on the core walls and bottoms. These experimental studies were conducted on sheep, pig and rabbits, including some juveniles and subadults, and some raw and some cooked samples^35,36^. While there are currently no human tooth marks in our comparative sample, based on the lack of other features of human tooth marks and chewing damage outlined in previous research, and the fact that human tooth marks have most often been identified on modern and fossil bones of smaller sized taxa and skeletal elements than this hominin tibia, we think it is possible but unlikely that the tooth marks on this fossil hominin tibia are hominin tooth marks.

The two BSM classified as tooth marks are most similar to those produced by a felid (Fig. 5). In our known sample of tooth marks, felids are only represented by the modern lion. While often considered to be hunters, lions are opportunistic in acquiring carcass foods and scavenge frequently. In wooded habitats lions kill between 78 and 88% of their food, but only kill about 47% of their food in open habitats^37^. This is most likely the result of an abundance of hyenas, from which lions frequently scavenge, and the visibility of vultures that can be followed to kills by lions in open habitats^37^. Lions will also scavenge from leopards, cheetahs, wild dogs, jackals, and when available, from animals that died from disease or malnutrition^37^. Given the variability in lion behavior and the presence of both cut marks and tooth marks on the fossil hominin tibia, it is not possible to infer the cause of death of the individual, or even the primary consumer. However, the location of the cut marks on the posterior tibia shaft suggests that flesh was likely on the carcass at the time that the cut marks were inflicted.

### Evidence for Pleistocene hominin butchery marks on hominins

There is uncontested evidence for cannibalism in European Neanderthals from sites in Belgium (Troisième Caverne of Goyet), France (Moula-Guercy and Padrelles), Spain (Cueva del Sidrón and Cueva del Boquete de Zafarraya), and Croatia (Krapina) (see ^24^^,^^25^ and references therein). There is also evidence for both anthropogenic defleshing and cannibalism in *Homo sapiens* from sites in Ethiopia (Herto), Poland (Maszycka Cave), the UK (Gough’s Cave), and possibly Germany (Brillenhöhle) (see^23^ and references therein). Yet there are still only a handful of Pleistocene sites with evidence for hominin cannibalism^24,25^, and only four published examples of postmortem defleshing on hominin fossils other than Neanderthals and *Homo sapiens*. We describe these examples in more detail here, in order from youngest to oldest.

Though slightly younger than the Early Pleistocene at ~ 600,000 years old, the first observation of cut marks on an early hominin was made by White^38^ on the Bodo cranium from the Middle Awash Valley of Ethiopia. White identified 17 areas with diagnostic cut marks on the Bodo cranium and used scanning electron microscopy to investigate some of the cut marks. He also studied crania of modern apes intentionally defleshed with steel knives during the early 1900s now housed at the Cleveland Museum of Natural History for comparison. He was able to match the placement and orientation of each set of the Bodo cut marks among these apes, despite differences in gross cranial morphology and tool type employed. He interpreted the marks on the Bodo cranium as patterned intentional postmortem defleshing of this specimen by a hominin with a stone tool^38^.

There is some evidence for processing on some of the at least 30 *Homo heidelbergensis* or *Homo erectus* individuals from Caune de l ‘Argo (also known as Arago Cave) in Tautavel, France, most dated to the ~ 680,000 year old Middle Pleistocene middle G and F levels. While a systematic taphonomic study of this site has not yet been undertaken, de Lumley^39^ described the remains, primarily from level G, as showing the presence of abundant cut marks on skulls and limb bones and green breakage of long bones. The difference in skeletal part profiles of hominins and non-hominin animals from Arago Cave led de Lumley to suggest that the hominin bones present at the site—skulls, mandibles, long limb bones, and a pelvic girdle—were anthropogenically selected and possibly related to ritualistic cannibalism. Cole^25^ noted that postcranial remains at this site were processed differently than cranial remains, also raising the possibility of ritual cannibalism. However, Saladié and Rodríguez-Hidalgo^24^ propose that the near absence of axial bones at Arago Cave could be the result of other processes, such as post-depositional destruction (which is more likely to affect these skeletal elements) or difficulties in taxonomic identification; they suggest that the taphonomic damage on the Arago hominins may not be due to ritual behavior.

Fernández-Jalvo et al.﻿^26,40^ first identified butchery marks among the ~ 772,000 to 949,000 year old *Homo antecessor* remains from Gran Dolina, which is part of the karstic site complex of the Sierra de Atapuerca in northern central Spain. The taphonomic analyses of human fossils from the TD6-Aurora Stratum of Gran Dolina, using a binocular light microscope as well as scanning electron microscopy, included identification not only of cut marks (including slicing marks, chop marks, and scraping marks), but also of percussion pits, peeling, conchoidal fracture, and adhering flakes. They found evidence of hominin-induced breakage, butchery marks, and human tooth marks on 44.5% of *Homo antecessor* bones, including many different skeletal elements, suggesting scalping, skinning, disarticulation, evisceration, and defleshing aimed at meat, marrow, and brain extraction^26^^,^^40^. The patterning of butchery damage on the *Homo antecessor* remains was generally similar to the patterning of damage on the non-human animal remains and was consistent with those bones that held the most nutritional value, as was the spatial distribution of human and non-human animal remains^26^^,^^40^. They found no evidence of ritual treatment and interpreted the butchery mark damage on the assemblage as evidence for nutritional cannibalism, more likely gastronomic cannibalism than survival cannibalism. Carbonell et al.^41^ described human meat consumption by *Homo antecessor* at this site as “frequent and habitual” and concluded that this nutritional cannibalism was accepted and included in their social system. After comparing the age profiles of the butchered *Homo antecessor* individuals to the age profiles seen in cannibalism associated with intergroup aggression in chimpanzees, Saladié et al.^42^ concluded that the Gran Dolina hominins periodically hunted and consumed individuals from another group. With evidence for processing of 11 individuals—2 adults, 3 adolescents, and 6 children^26^—this is arguably the earliest firm evidence of systematic cannibalism in the hominin fossil record, and the only such evidence from the Early Pleistocene.

In 2000, Pickering et al.^43^ published what are currently accepted as the oldest cut marks on a hominin fossil: cut marks inflicted by a stone tool on the right maxilla of Stw 53, a partial skull from Sterkfontein Member 5 (or possibly a hanging remnant of Member 4—an area called the STW 53 infill) in South Africa. This skull was first found in 1976 by Alun Hughes and is generally attributed to *Homo habilis* but is also sometimes argued to represent *Australopithecus*. Pickering et al. noted that the morphology of the marks, their anatomical placement, and the lack of random striae on the specimen all support an interpretation of this linear damage as cut marks^43^. They state that the location of the marks on the lateral aspect of the zygomatic process of the maxilla is consistent with that expected from slicing through the masseter muscle, presumably to remove the mandible from the cranium, in an act of disarticulation. The age of this fossil is somewhat uncertain; Pickering and Kramers^44^ suggest an age between 2.6 and 2.0 Ma, but Wood^20^ thinks it is younger, between 2.0 and 1.5 Ma. A more recent reassessment of these marks by Hanon et al.^45^ including macro- and microscopic observations concluded that they are not evidence for hominin butchery, but the result of natural processes. They noted that Clarke^46^ mentioned that the zygomatic bone of Stw53 was discovered with sharp-edged chert blocks lying against it, which could produce linear marks under sedimentary pressure, and assert that the morphology of these marks is more consistent with trampling marks. Because the marks are located on the masseter muscle insertion of the zygomatic maxillary process they also looked for corresponding marks on the masseter muscle insertion area of the temporal bone, which might be expected in the process of disarticulation or defleshing, but did not find any. While this more recent analysis of the purported cut marks on Stw 53 has not been replicated or confirmed, given the uncertain age of Stw 53, KNM-ER 741 is now at least among the oldest hominin fossils with evidence of hominin butchery marks, and currently is the oldest known hominin butchery marked postcranial fossil.

**Figure 6:**
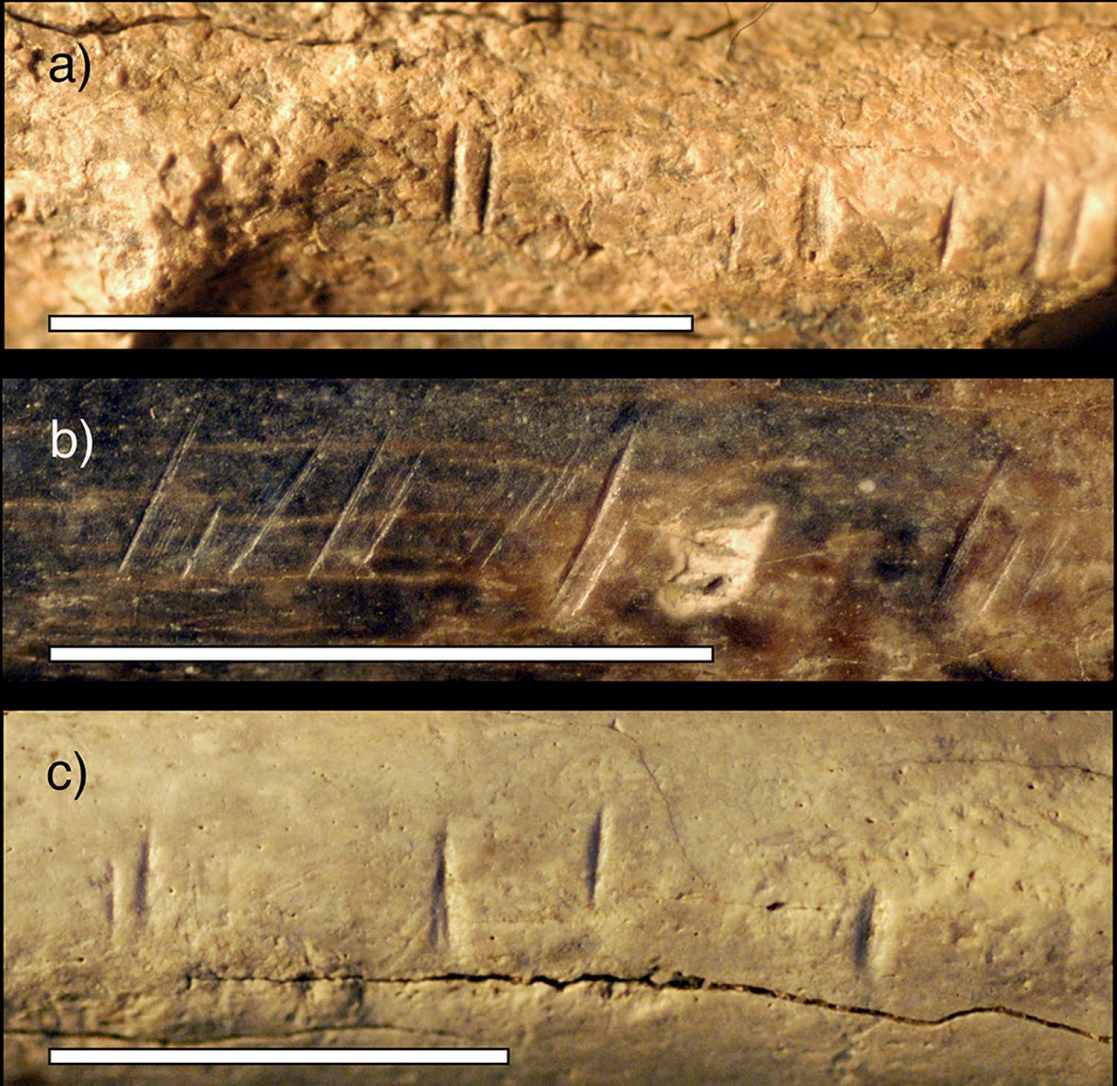
Close up photos of three fossil fauna specimens from archaeological surface finds and excavations in the Okote Member of Koobi Fora (Pobiner^47^), showing similar cut marks to those found on KNM-ER 741. (**a**) FwJj14B 5097, a bovid size 3 mandible with cut marks on the inferior margin, found in situ (**b**) FwJj14A 1016-97, a bovid size 3 radius midshaft with cut marks, found on the surface (**c**) GaJj14 1056, a large mammal scapula with cut marks along scapular margin, found on the surface. Scale = 1 cm.

Previous research on butchery marked fauna from multiple sites from the Okote Member of Koobi Fora show that at this time (~ 1.5 million years ago), hominins were consistently using stone tools to deflesh, disarticulate, and extract marrow from a variety of species and sizes of animals from different habitat settings^13–15^. While the morphology of the butchery marks on the Okote Member sites is variable^47^, some generally resemble the marks on KNM-ER 741 (Fig. 6). Quantitative analyses of the bone surface modifications on the KNM-ER 741 tibia demonstrate a high level of confidence that the majority were produced by hominins. But what was being cut, exactly, on KNM-ER 741—and why? The cut marks are located on the posterio-medial border of the proximal tibia shaft, near the origin of the popliteus muscle, which rotates the knee medially and flexes the knee joint. The popliteus origin is situated beneath the gastrocnemius, which would have had to be removed for access to the cut marked area. We interpret the location of the cut marks to be more likely the result of defleshing than disarticulation. We conclude that if anthropophagy occurred after the defleshing of KNM-ER 741, it was an opportunistic, practical, and functional activity which occurred simply in the context of obtaining food, rather than one imbued with ritual meaning.

## Methods

Pobiner used a 10X hand lens and bright, high-incident light to look for bone surface modifications on hominin postcranial bones at the National Museums of Kenya, including KNM-ER 741, following methods outlined in Blumenschine et al.^48^. She used Coltene President Plus light body dental molding material to create an impression of the marks analyzed in this study.

The quantitative analysis of bone surface modifications (BSM) followed the protocol presented in Pante et al.^49^. 3-D models of each of the 11 studied BSM were created from an impression of the surface taken from the area of interest on the tibia using a Nanovea ST400 white-light non-contact confocal profilometer equipped with a 3 mm optical pen (objective) that has a resolution of 40 nm on the z-axis. The resolution on the x-axis was set to 5 um and 10 um on the y-axis. Processing and analysis of the 3D model was carried out using Digital Surf's Mountains^®^ following Pante et al.^49^. Processing included removing outliers, filling in missing data points, and removing the underlying form of the bone. Data collected through the analysis from the entire 3-D model of the BSM were volume, surface area, maximum depth, mean depth, maximum length, and maximum width. Additional data were collected from a profile taken from the deepest point of the BSM including area of the hole, depth of the profile, roughness (Ra), opening angle, and radius of the hole.

These data were statistically compared with a sample of 898 BSM of known origin, including: 402 cut marks from a variety of stone tool types and raw materials; 278 tooth marks from crocodiles and five species of mammalian carnivores; 130 trample marks produced by cows on substrates including sand, gravel, and soil; and 88 percussion marks from both anvils and hammerstones (see Supplemental document 1 for details on the model structure, influential variables, canonical data, and box-cox transformation values). Surface area and depth of the profile were excluded from the statistical analyses because they are highly correlated with volume and maximum depth, respectively, which can lead to overfitting of data. All experimental data were transformed using the Box-Cox method to normalize the distributions for each variable and the same transformations were applied to the archaeological data. Comparisons were carried out using the quadratic discriminant function in JMP® statistical software. The accuracy of the quadratic function in correctly classifying the experimental BSM was 84% when using a 25% validation set. Prior probabilities were set equal to the occurrence of each mark type in the dataset to offset the disproportionate representations of each mark type in the experimental sample.

## Supplementary Information

Supplementary Information.

## Data Availability

All study data are included in the article.

## References

[1] Hart D, Sussman RW, Sussman RW, Cloninger CR (2011). The influence of predation on primate and early human evolution: impetus for cooperation. Origins of Altruism and Cooperation.

[2] Leakey MG, Feibel CS, McDougall I, Ward C, Walker A (1998). New specimens and confirmation of an early age for *Australopithecus*
*anamensis*. Nature.

[3] Ward CV, Leakey MG, Walker AC (1999). The new hominid species *Australopithecus*
*anamensis*. Evol. Anth..

[4] Pickering TR, Clarke RJ, Moggi-Cecchi J (2004). Role of carnivores in the accumulation of the Sterkfontein Member 4 hominid assemblage: A taphonomic reassessment of the complete hominid fossil sample (1936–1999). Am. J. Phys. Anth..

[5] Johanson DC, Lovejoy CO, Kimbel WH, White TD, Ward SC, Bush ME, Latimer BM, Coppens Y (1982). Morphology of the Pliocene partial hominid skeleton (A.L. 288-1) from the Hadar formation, Ethiopia. Am. J. Phys. Anth..

[6] Kappelman J, Ketcham RA, Pearce S, Todd L, Akins W, Colbert MW, Davis C, Feseha M, Maisan JA, Witzel A (2018). Reply to: Charlier et al. 2018. Mudslide and/or animal attack are more plausible causes and circumstances of death for AL 288 (‘Lucy’): a forensic anthropology analysis. Medico-Legal Journal 86(3) 139–142, 2018. Med.-Legal J..

[7] Brain CK (1981). The Hunters or the Hunted. An Introduction to African Cave Taphonomy.

[8] Njau JK, Blumenschine RJ (2012). Crocodylian and mammalian carnivore feeding traces on hominid fossils from FLK 22 and FLK NN 3, Plio-Pleistocene, Olduvai Gorge, Tanzania. J. Hum. Evol..

[9] Pobiner BL (2020). The zooarchaeology and paleoecology of early hominin scavenging. Evol. Anth..

[10] Barr WA, Pobiner B, Rowan J, Du A, Faith JT (2022). No sustained increase in zooarchaeological evidence for carnivory after the appearance of *Homo*
*erectus*. Proc. Natl. Acad. Sci..

[11] Plummer TL, Oliver JS, Finestone EM, Ditchfield PW, Bishop LC, Blumenthal SA, Lemorini C, Caricola I, Bailey S, Herries AIR, Parkinson JA, Whitfield E, Hertel F, Kinjanjui RN, Vincent TH, Li Y, Louys J, Frost SR, Braun DR, Reeves JS, Early EDG, Onyango B, Lamela-Lopez R, Forrest FL, He H, Lane TP, Frouin M, Nomade S, Wilson EP, Bartilol SK, Rotich NK, Potts R (2023). Expanded geographic distribution and dietary strategies of the earliest Oldowan hominins and *Paranthropus*. Science.

[12] Espigares MP, Palmqvist P, Guerra- Merchán A, Ros-Montoya S, García-Aguilar JM, Rodríguez-Gómez G, Serrano FJ, Martínez-Navarro B (2019). The earliest cut marks of Europe: A discussion on hominin subsistence patterns in the Orce sites (Baza basin, SE Spain). Sci. Rep..

[13] Pobiner BL, Rogers MJ, Monahan CM, Harris JWK (2008). New evidence for hominin carcass processing strategies at 1.5 Ma, Koobi I, Kenya. J. Hum. Evol..

[14] Merritt SR (2017). Investigating hominin carnivory in the Okote Member of KoIFora, Kenya with an actualistic model of carcass consumption and traces of butchery on the elbow. J. Hum. Evol..

[15] Merritt SR, Mavuso S, Cordiner EA, Fetchenhier K, Greiner E (2018). FwJj70: A potential early stone age single carcass butchery locality preserved in a fragmentary surface assemblage. J. Arch. Sci. Rep..

[16] Leakey MG, Leakey RE, editors. Koobi Fora Research Project Volume 1: The Fossil Hominids and an Introduction to Their Context, 1968-1974. Oxford University Press; 1977. 191 pp.16.

[17] Leakey RE (1971). Further evidence of lower Pleistocene hominids from East Rudolf, North Kenya. Nature.

[18] Leakey RE (1973). Further evidence of lower Pleistocene hominids from East Rudolf, North Kenya, 1972. Nature.

[19] Walker A, Leakey R, editors. The Nariokotome *Homo Erectus* Skeleton. Berlin Heidelberg: Springer-Verlag; 1993. 458 pp.

[20] Wood B (2011). Wiley-Blackwell Encyclopedia of Human Evolution.

[21] Domínguez-Rodrigo M, de Juana S, Galán AB, Rodríguez M (2019). A new protocol to differentiate trampling marks from butchery cut marks. J. Arch. Sci..

[22] Fernández-Jalvo Y, Andrews P (2016). Atlas of Taphonomic Identifications. 1001+ Images of Fossil and Recent Mammal Bone Modification.

[23] Cole J (2017). Assessing the calorific significance of episodes of human cannibalism in the Palaeolithic. Sci. Rep..

[24] Saladié P, Rodríguez-Hidalgo A (2017). Archaeological evidence for cannibalism in prehistoric western Europe: From *Homo*
*antecessor* to the Bronze Age. J. Archaeol. Method Theory.

[25] Cole J. Consuming passions: Reviewing the evidence for cannibalism within the Prehistoric archaeological record [Internet]. Ass–mblage: Sheff. Grad. J. Archaeol. **9** (2006).

[26] Fernández-Jalvo Y, Carlos Díez J, Cáceres I, Rosell J (1999). Human cannibalism in the Early Pleistocene of Europe (Gran Dolina, Sierra de Atapuerca, Burgos, Spain). J. Hum. Evol..

[27] Turner CGI, Turner JA (1999). Man Corn: Cannibalism and Violence in the Prehistoric American Southwest.

[28] Knüsel C, Outram A (2006). Fragmentation of the body: Comestibles, compost, or customary rite?. Soc. Archaeol. Funer. Remains.

[29] White T (1992). Cannibalism at Mancos 5MTUMR-2346.

[30] Tutt CMA (2003). Cannibalism among fossil hominids: Is there archaeological evidence?. Totem: Univ. West Ontario J Anth..

[31] Fernández-Jalvo Y, Andrews P (2021). Butchery, art or rituals. J. Anthro. Archeo. Sci..

[32] Villa P, Bouville C, Courtin J, Helmer D, Mahieu E, Shipman P (1986). Cannibalism in the Neolithic. Science.

[33] Saladié P, Cáceres I, Huguet R, Rodríguez-Hidalgo A, Santander B, Ollé A (2015). Experimental butchering of a chimpanzee carcass for archaeological purposes. PLoS ONE.

[34] Bello SM, Wallduck R, Dimitrijević V, Živaljević I, Stringer CB (2016). Cannibalism versus funerary defleshing and disarticulation after a period of decay: Comparisons of bone modifications from four prehistoric sites. Am. J. Phys. Anthropol..

[35] Fernández-Jalvo Y, Andrews P (2011). When humans chew bones. J. Hum. Evol..

[36] Saladié P, Rodríguez-Hidalgo A, Díez C, Martín-Rodríguez P, Carbonell E (2013). Range of bone modifications by human chewing. J. Arch. Sci..

[37] Schaller GB (1972). The Serengeti Lion: A Study of Predator Prey Relations.

[38] White TD (1986). Cut marks on the Bodo cranium: A case of prehistoric defleshing. Am. J. Phys. Anthropol..

[39] de Lumley M-A (2015). L’homme de Tautavel. Un Homo erectus européen évolué *Homo*
*erectus* tautavelensis. L’Anthropologie.

[40] Fernández-Jalvo Y, Díez JC, de Castro JMB, Carbonell E, Arsuaga JL (1996). Evidence of early cannibalism. Science.

[41] Carbonell E, Cáceres I, Lozano M, Saladié P, Rosell J, Lorenzo C (2010). Cultural cannibalism as a paleoeconomic system in the European Lower Pleistocene. Curr. Anthropol..

[42] Saladié P, Huguet R, Rodríguez-Hidalgo A, Cáceres I, Esteban-Nadal M, Arsuaga JL (2012). Intergroup cannibalism in the European Early Pleistocene: The range expansion and imbalance of power hypotheses. J. Hum. Evol..

[43] Pickering TR, White TD, Toth N (2000). Brief communication: Cutmarks on a Plio-Pleistocene hominid from Sterkfontein, South Africa. Am. J. Phys. Anthropol..

[44] Pickering R, Kramers JD (2010). Re-appraisal of the stratigraphy and determination of new U-Pb dates for the Sterkfontein hominin site, South Africa. J. Hum. Evol..

[45] Hanon R, Péan S, Prat S (2018). Reassessment of anthropic modifications on the Early Pleistocene hominin specimen Stw53 (Sterkfontein, South Africa). Bull. Mém Soc. Anthropol. Paris.

[46] Clarke R. Australopithecus from Sterkfontein Caves, South Africa. In: *The Paleobiology of Australopithecus*. 2013. pp 105–23.

[47] Pobiner B. *Hominin-carnivore interactions: evidence from modern carnivore bone modification and Early Pleistocene archaeofaunas (Koobi Fora, Kenya; Olduvai Gorge, Tanzania).* 2007; PhD Dissertation, Rutgers University.

[48] Blumenschine RJ, Marean CW, Capaldo SD (1996). Blind tests of inter-analyst correspondence and accuracy in the identification of cut marks, percussion marks, and carnivore tooth marks on bone surfaces. J. Archaeol. Sci..

[49] Pante MC, Muttart M, Keevil T, Blumenschine RJ, Njau JK, Merritt SM (2017). A new high-resolution 3-D quantitative method for identifying bone surface modifications with implications for the Early Stone Age archaeological record. J. Hum. Evol..

